# Immune checkpoint inhibitors in metastatic melanoma therapy (Review)

**DOI:** 10.3892/mi.2024.137

**Published:** 2024-02-09

**Authors:** Vedant Shah, Viraj Panchal, Abhi Shah, Bhavya Vyas, Siddharth Agrawal, Sanket Bharadwaj

**Affiliations:** Department of Medicine, Smt. N.H.L. Municipal Medical College and Sardar Vallabhbhai Patel Institute of Medical Sciences and Research (SVPISMR), Ahmedabad, Gujarat 380058, India

**Keywords:** ipilimumab, nivolumab, pembrolizumab, melanoma, immune checkpoint inhibitors

## Abstract

An increase in the incidence of melanoma has been observed in recent decades, which poses a significant challenge due to its poor prognosis in the advanced and metastatic stages. Previously, chemotherapy and high doses of interleukin-2 were available treatments for melanoma; however, they offered limited survival benefits and were associated with severe toxicities. The treatment of metastatic melanoma has been transformed by new developments in immunotherapy. Immune checkpoint inhibitors (ICIs), monoclonal antibodies that target cytotoxic T-lymphocyte-associated antigen-4 (CTLA-4), programmed cell death protein 1 (PD-1) and its ligand, PDL-1, have emerged as promising therapeutic options. Commonly used ICIs, such as ipilimumab, nivolumab and pembrolizumab, have been found to be associated with an improved median overall survival, recurrence-free survival and response rates compared to traditional chemotherapies. Combination therapies involving different types of ICIs, such as anti-PD1 with anti-CTLA-4, have further enhanced the overall survival and response rates by targeting various phases of T-cell activation. Additionally, the development of novel biomarkers has facilitated the assessment of responses to ICI therapy, with tissue and serum-based prognostic and predictive biomarkers now available. The increased response observed with ICIs also provides potential for immune-related adverse effects on various organ systems. Further research is required to evaluate the efficacy and safety of various combinations of ICIs, while ongoing clinical trials explore the potential of newer ICIs. Concerns regarding the development of resistance to ICIs also warrant attention. The present review summarizes and discusses the advent of ICIs with a marked significant breakthrough in the treatment of metastatic melanoma, providing improved outcomes compared to traditional therapies.

## 1. Introduction

The incidence of melanoma has increased over the past two decades (1). Each year, melanoma affects more than 325,000 people. Males experience more frequent occurrences, with 174,000 yearly cases compared to females, with 151,000 cases (2). Of note, 20% of patients with melanoma eventually develop unresectable or distant metastatic disease labeled as stage III/IV (3). Unfortunately, for such an advanced-stage disease, the prognosis remains bleak. However, understanding the advanced stages of melanoma growth and progression has led to the development of promising new therapeutic alternatives.

Until 2011, chemotherapy was the initial treatment for metastatic melanoma; however, it only provided a 6-month median survival time and a 25% 1-year overall survival rate. High-dose interleukin-2 (IL-2) was the only immunotherapy available, but was associated with severe toxicities and only benefited a limited number of patients (4). Currently, advances in immunotherapy and studies on cell cycle regulatory molecules have facilitated the creation of immune checkpoint inhibitors (ICIs), a group of monoclonal antibodies that block co-inhibitory molecules, such as cytotoxic T-lymphocyte-associated antigen-4 (CTLA-4), programmed cell death protein 1 (PD-1) and its ligand, PDL1 (5-7). Ipilimumab, nivolumab and pembrolizumab were the first class of medications shown to improve the overall survival of patients with metastatic melanoma (4).

The present review provides comprehensive evidence regarding the role of ICIs and their utilization in advanced melanoma cases. The outcomes of ICIs, such as ipilimumab, nivolumab and pembrolizumab are highlighted, including the improved survival rates and response rates associated with their use compared to traditional chemotherapies, while also focusing on the mechanisms and demonstrating the potentially adverse effects of these therapies.

Furthermore, the present review focuses on combination therapies, including anti-PD1 with anti-CTLA-4, showcasing their importance compared to monotherapy. The improved outcomes of combination therapies over traditional therapies are highlighted, with an emphasis on the need for ongoing research, optimized treatment approaches and strategies which can be used to overcome resistance. In addition to discussing the development of novel biomarkers for assessing ICI therapeutic responses in both tissue and serum-based prognostic and predictive markers, tumor metabolic dependencies and targeting the metabolic pathways by combining ICIs are also discussed. This could provide an improved efficacy, which, to the best of our knowledge, has not been described commonly in the available literature focusing on ICIs used in melanoma.

## 2. Immune checkpoint inhibitor drugs and their role in metastatic melanoma

**Figure 1:**
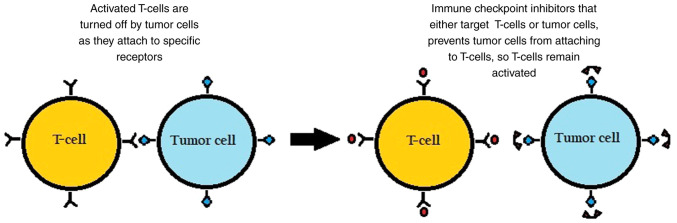
Illustration demonstrating the role of immune checkpoint inhibitors in suppressing the activation of T-cells or preventing the release of cytokines from tumor cells.

The surface of immune cells, such as T-cells, assists in the regulation of the immune response through various receptors. When activated by certain ligands, these receptors inhibit immune cells from attacking the body's own cells. However, in cancer, tumor cells can take advantage by binding to these checkpoint-inhibitory receptors through their own ligands and suppressing the immune response, as illustrated in Fig. 1.

**Figure 2:**
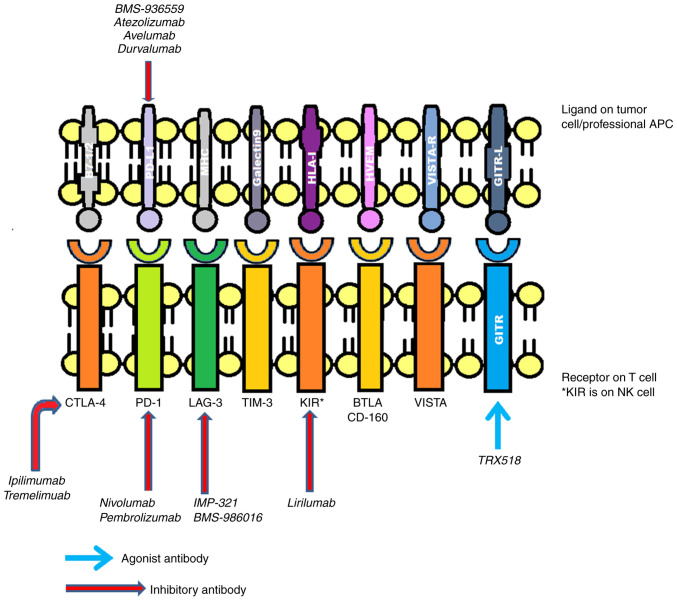
Various types of immune receptors on T-cells and their interaction with ligands present on tumor cells or immune checkpoint inhibitors in the treatment of melanoma. APC, antigen-presenting cell; CTLA-4, cytotoxic T-lymphocyte-associated antigen-4; PD-1, programmed cell death protein 1; LAG-3, lymphocyte-activation gene-3; TIM-3, T-cell immunoglobulin domain and mucin domain-3; BTLA, B- and T-lymphocyte attenuator; KIR, killer cell immunoglobulin-like receptor; VISTA, V-domain immunoglobulin (Ig)-containing suppressor of T-cell activation; NK cell, natural killer cell.

Commonly implicated inhibitory receptors include (CTLA-4, PD-1, T-cell immunoglobulin domain and mucin domain-3 (TIM-3), killer cell immunoglobulin-like receptor (KIR), lymphocyte-activation gene 3 (LAG3), glucocorticoid-induced tumor necrosis factor receptor (GITR), B- and T-lymphocyte attenuator (BTLA) and V-domain immunoglobulin (Ig)-containing suppressor of T-cell activation (VISTA) (8-10), as presented in Fig. 2.

Over time, several drugs have been introduced targeting these receptors. Ipilimumab was one of the first ICI drugs to be approved by the Food and Drug Administration (FDA) for the treatment of metastatic melanoma, which functions by blocking CTLA-4(11). There are numerous additional comparable drugs in early phase III, phase II, or preclinical research. These include pidilizumab, atezolizumab, durvalumab and tremelimumab (formerly known as ticilimumab) (12,13). To increase the immune system's defense against cancer cells, these medications also work against various immunological checkpoints (12,13).

## 3. Mechanisms underlying the effectiveness of ICIs in metastatic melanoma

### CTLA-4 and anti-CTLA-4 drugs

CTLA-4, a B7/CD28 family member, is a coinhibitory receptor expressed on the surface of T-cells that eventually inhibits T-cells, and it is expressed by regulatory T-cells (Tregs) (14). Discovered in 1987, it was considered to function as a negative regulator of T-cell activation until the mid-1990s (15-17).

**Figure 3:**
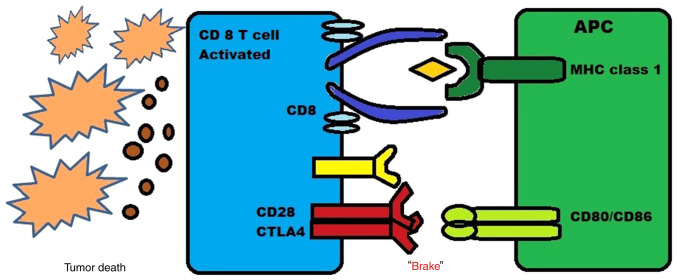
Illustration demonstrating the interaction of CTLA-4 expression on T-cells and CD80 and CD86 on APCs. CTLA-4, cytotoxic T-lymphocyte-associated antigen-4; APC, antigen-presenting cell; MHC, major histocompatibility complex.

When CTLA-4 is expressed on the surface of CD4^+^ and CD8^+^ T-cells, the binding affinity increases and is higher to CD80 and CD86, which are the costimulatory receptors present on antigen-presenting cells (APCs) compared to CD28 which is another costimulatory receptor (18). The expression of CTLA-4 increases when there is an activation of T-cell receptors and the release of cytokines such as IL-12 and interferon (IFN)-γ. This upregulation creates a feedback inhibition loop on T-effector cells which are activated, leading to CTLA-4 acting as a natural ‘brake’ on CD4^+^ and CD8^+^ T-cell activation induced by APCs, as shown in Fig. 3.

Tregs also play a key role in maintaining immune homeostasis by inhibiting excessive immune responses. One of the mechanisms through which Tregs suppress effector T-cell activity is via CTLA-4 signaling (19). Two anti-CTLA-4 drugs have been studied in patients with melanoma: i) Ipilimumab, the first ICI evaluated and approved for the treatment of melanoma is a fully human immunoglobulin anti-CTLA-4 monoclonal antibody (20,21); ii) tremelimumab, a fully human immunoglobulin anti-CTLA-4 monoclonal antibody which is still under investigation (12).

There are two major mechanisms through which these drugs act. First, the inhibition of CTLA-4 signaling in cytotoxic T-cells that specifically target tumors can directly affect these cells by enabling them to evade a state of anergy and enter an active proliferative effector phase. Once activated, these effector T-cells are more likely to penetrate the tumor and exhibit direct cytotoxic effects on tumor cells, while also releasing cytokines such as IL-2 and IFN-γ to stimulate an immunogenic tumor microenvironment. Thus, by blocking the CTLA-4 pathway, T-cells that were previously inactive can become activated and effectively target the tumor cells, causing a more powerful immune response against the cancer. This new approach holds promise as a potential immunotherapy for the treatment of cancer (22).

The second major mechanism driving these drugs is the blocking of CTLA-4 signaling in Tregs, which may impair their ability to halt the activity of effector T-cells. This inhibition of CTLA-4 signaling can either cause a decrease in the number of Tregs or reduce their function without affecting their population size. Therefore, blocking CTLA-4 on Tregs may disrupt this suppression and lead to increased immune activation against tumor cells (19,23,24).

Patients with melanoma are treated with the primary aim of suppressing the molecular interplay between the melanoma cells and immune effector cells. Ipilimumab, which has mainly been approved for the treatment of more advanced stages, such as unresectable or metastatic melanoma, has been shown to be associated with a marked overall survival rate confirmed from a phase 3 clinical trial (25). The interference of ipilimumab on CTLA-4 expressed on the subset of tumor-specific T-cell proliferation and B7 molecules on antigen-presenting cells is expected to prevent tumor development (26).

### PD-1 pathway and anti-PD-1/PD-L1 drugs

**Figure 4:**
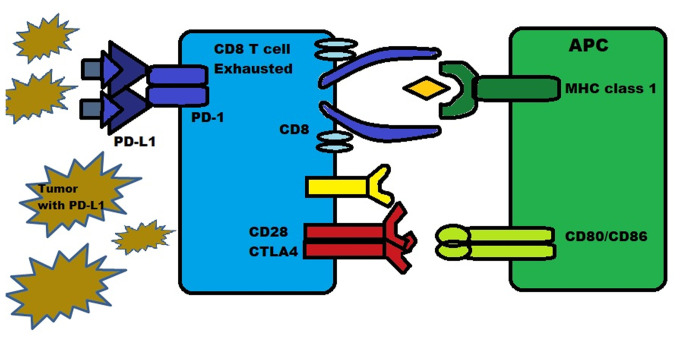
Illustration demonstrating the interaction between PD-1 and PD-L1 among T-cells and tumor cells. PD-1, programmed cell death protein 1; PD-L1, programmed cell death protein ligand 1; CTLA-4, cytotoxic T-lymphocyte-associated antigen-4; MHC, major histocompatibility complex.

PD-1 is a protein present on the surface of T-cells, B-cells and natural killer (NK) cells. It functions as an inhibitory molecule by binding to PD-L1 (or B7-H1) and PD-L2 (B7-H2). PD-L1 is expressed in numerous types of tissue, including hematopoietic cells and certain tumors such as melanoma, where they are expressed in 40-50% of cases. PD-L2 is mainly expressed in hematopoietic cells. The binding of PD-1 to PD-L1/2 inhibits the death of tumor cells and promotes the conversion of T-effector cells into Tregs, while also inducing exhaustion in peripheral T-effector cells, as illustrated in Fig. 4 (27,28).

PD-1 and/or PD-L1 are also expressed on cells, such as NK cells, monocytes and dendritic cells (27,28). The PD-1 pathway operates through various mechanisms, such as reducing the activity of T-cells during an inflammatory response, increasing the proliferation and suppressive activity of Tregs, and reducing the lytic activity of B-cells and NK cells (29).

The affinity between PD-1 and PD-L1 is 3-fold stronger than the affinity between PD-1 and PD-L2. When PD-L1 binds with PD-1 on T-cells, it results in T-cell exhaustion, dysfunction, neutralization and the production of IL-10 within the tumor mass. This process allows tumors that overexpress PD-L1 to protect themselves from being attacked and killed by CD8^+^ cytotoxic T-cells (30).

Pro-effector cytokines, namely IL-12 and IFN-γ, can upregulate the expression of PD-1 and PD-L1/L2, which helps to prevent excessive T-effector cell activity. It is worth noting that PD-L1 has also been shown to inhibit CD80, indicating the existence of complex interactions between CTLA-4, PD-1 and other pathways (31,32).

The PD-1 and PD-L1 antibody inhibitors were created with the aim of preventing the PD-1 or PD-L1 side from functioning, reactivating T-cells and promoting an immune response against cancer cells (13).

Based on promising results from clinical trials, antibodies that inhibit PD-1 (such as pembrolizumab, nivolumab-IgG4 fully humanized and dostarlimab), as well as those that inhibit PD-L1 (such as avelumab, atezolizumab, and durvalumab) are being evaluated for use in melanoma cases and various other malignancies (NCT04020809, NCT04274816, NCT03313206, NCT03842943 and NCT05928962). However, it is not yet known which inhibitor, PD-1 or PD-L1, is more efficient (13). Of these, nivolumab and pembrolizumab are the two major FDA-approved anti-PD-1 monoclonal antibodies available for the treatment of advanced and metastatic melanomas.

Melanoma cells exhibit increased levels of PD-L1, which promotes the apoptosis of the likewise increased levels of T-cells (33). It has also been found that the circulating melanoma antigen-specific T-cells and tumor-infiltrating lymphocytes express PD-1 abnormally. It is considered that melanoma cells are capable of initiating, as well as sustaining PD-1 signals, T-cell fatigue and dysfunction (33). Hence, by blocking PD-1 in patients with melanoma, one could possibly restore abnormal activation and signaling and eventually recover the immune effect. Pembrolizumab or lambrolizumab were used in unresectable or metastatic melanoma in the study by Hamid *et al* (34), in an aim to elucidate the effects of PD-1 medications in melanoma.

However, due to the heterogeneous nature of tumors, the expression of PD-L1 is not uniform throughout. The extent of PD-L1 expression can differ in various locations within the tumor, resulting in varying levels of PD-L1 in immunohistochemical staining. Moreover, the effectiveness of PD-L1/PD-1 inhibitors can also be influenced by several other factors, such as the type of cancer, the patient's immune system and the genetic profile of the tumor. Thus, a more in-depth understanding of these factors is essential for developing effective treatment strategies that consider the heterogeneity of tumors and the variability in PD-L1 expression (35).

The combination ICI therapy used in the treatment of patients with metastatic melanoma primarily involves CTLA-1 and PD-1 inhibitors. This amplified the inhibitions that can be simultaneously intervened during different phases of the interaction among melanoma cells and the immune system. This, for example, includes anti-CTLA-4 inhibiting the priming stage at the same time anti-PD-1 inhibits the effector stage (36,37). It has also been noted that the use of anti-CTLA-4 inhibitors results in an increased expression of PD-1; hence, using combination therapy results in a more robust treatment response in patients with melanoma (38).

### Newer drugs

An increased understanding of immunological mechanisms has led to the identification of additional potential targets for checkpoint inhibition in the treatment of cancer. Some of these potential targets include BTLA, VISTA, TIM-3, CD47 and LAG-3. i) The blockade of BTLA has been shown to enhance New York esophageal squamous cell carcinoma 1) specific CD8^+^ T-cell function and enhance the efficacy of anti-PD-1 (39-41). ii) VISTA blockade has been shown to increase T-cell infiltration and function in tumors, thereby reducing tumor growth (10,42). iii) TIM-3 blockade causes T-helper-1 cell hyperproliferation and cytokine release, leading to tumor shrinkage in a mouse model when combined with anti-CTLA-4 or anti-PD-1 (43-47). iv) Targeting CD47 with a humanized anti-CD47 monoclonal antibody in combination with rituximab has shown to lead to objective responses in half of the heavily pretreated patients with relapsed or refractory non-Hodgkin's lymphoma, including a complete response in more than one-third of patients (48). v) An immune pathway known as LAG-3 has been identified as a potential complement to the PD-1/PDL1 pathway in enhancing the immune response against cancer. LAG-3 is an immune checkpoint receptor that regulates the function of T-cells. BMS-986016 is a therapy that targets LAG-3 and is currently under investigation in combination with nivolumab, which targets PD-1, to enhance the immune response against cancer cells. The combination of these two therapies has the potential to create a synergistic effect, leading to improved treatment outcomes for patients with cancer (49).

## 4. Combination therapy with immune checkpoint inhibitors in melanoma

The ability of anti-CTLA-4 and anti-PD-1/PD-L1 monoclonal antibodies to target various T-cell activation locations and phases is the rationale for their combined use. PD-1 is primarily expressed on antigen-experienced T-cells in peripheral tissues, while CTLA-4 is expressed by naive T-cells in the lymph nodes. According to pre-clinical research, combining ICIs is more effective than treatment with with monotherapy for managing melanoma (50-53).

In pre-clinical investigations, anti-CTLA-4 and anti-PD-1/PD-L1 monoclonal antibodies have been shown to induce the infiltration of CD8^+^ T-cells and the expansion of an inducible T-cell co-stimulator (ICOS)^+^ T helper 1-like CD4 fraction, which in turn induces the response of CD4^+^ effector T-cells. Based on this, the sequencing or combination of nivolumab with ipilimumab in metastatic cutaneous melanoma has been researched (50-53). Other combination studies are on nivolumab, relatlimab and combination therapy with pembrolizumab with low-dose ipilimumab (54,55). The data from the CheckMate and RELATIVITIY047 trials on the combination of ICIs are presented in Table I, which demonstrate a favorable response for such therapies (54,56,57). Table II presents data from a meta-analysis, comparing monotherapy and combination therapy (47-64).

## 5. Comparison between the immune checkpoint inhibitors

Along with the major breakthrough in melanoma treatment with the use of selective BRAF inhibitors, after ~6 months of the median duration, resistance to therapy began to develop. The BRAF mutation drives the tumor proliferation exponentially by activating mitogen-activated kinase pathway (MAP), and the development of resistance to BRAF inhibitors in both MAP kinase-dependent and MAP kinase-independent pathways (65-67). Resistance in MAP kinase-dependent pathways includes secondary mutations in NRAS, the increased expression of COT kinase, CRAF activation and acquired mutations in MEK1 (65,68-71). MAP kinase-independent pathways include the upregulation of platelet-derived growth factor receptor, additional receptor tyrosine kinases activation including AXL, Erb-B2 receptor tyrosine kinase 4) and insulin like growth factor 1 receptor, the activation of PI3K/AKT signaling, and the loss of phosphatase and tensin homolog (PTEN) (65,69,71-75).

### Ipilimumab

Ipilimumab is a fully human monoclonal antibody developed to antagonize CTLA-4. A clinical study was conducted on patients who had unresectable stage III or IV melanoma, where they were randomly assigned in a 3:1:1 ratio to receive ipilimumab (3 mg/kg) plus the glycoprotein 100 (gp100) vaccine, ipilimumab alone, or gp100 alone. The patients who received ipilimumab plus gp100 had a longer median overall survival rate of 10 months compared to 6.4 months for those who received gp100 alone, with a hazard ratio (HR) for mortality of 0.68 and a statistically significant P-value of <0.001. The median overall survival rate of patients who received ipilimumab alone was 10.1 months (HR, 0.66; P=0.003) compared to those who received gp100 alone (20).

In the study by Robert *et al* (25), patients with untreated metastatic melanoma were randomly assigned to receive either ipilimumab plus dacarbazine or dacarbazine alone. A significantly longer median overall survival (11.2 months) was found in the ipilimumab plus dacarbazine group compared to the dacarbazine alone group (9.1 months). In a follow-up maintenance study, patients who received ipilimumab plus dacarbazine had a higher 5-year survival rate (18.2%) compared to those who received dacarbazine alone (8.8%). These findings suggest that ipilimumab in combination with dacarbazine may be an effective treatment option for metastatic melanoma patients (76).

In a trial for ipilimumab as an adjuvant treatment performed by Eggermont *et al* (77), patients with stage III cutaneous melanoma who had undergone complete resection were randomly administered either ipilimumab (10 mg/kg) (n=475) or a placebo (n=476). Following a median follow-up of 5.3 years, the ipilimumab group had a 5-year recurrence-free survival rate of 40.8%, while the placebo group had a rate of 30.3% (HR, 0.76; P<0.001). In terms of the 5-year overall survival rate, the ipilimumab group had a rate of 65.4% compared to 54.4% in the placebo group (HR, 0.72; P=0.001) (77).

In the study by Ascierto *et al* (78), patients with unresectable stage III or IV melanoma were randomly assigned to receive either 10 or 3 mg/kg ipilimumab. The median overall survival rate was 15.7 months for the 10 mg/kg group and 11.5 months for the 3 mg/kg group (HR, 0.84; P=0.04). Overall, their study suggested that ipilimumab treatment improved the survival outcomes of patients with unresectable stage III or IV melanomas, with higher doses of the drug (10 mg/kg) leading to an improved overall survvial compared to lower doses (3 mg/kg) (78).

### Nivolumab

Nivolumab is a monoclonal antibody that inhibits the interaction of PD-1 with PD-L1. The clinical study performed by Robert *et al* (79) compared the efficacy of nivolumab with the standard therapy of dacarbazine. In their study, patients who had metastatic melanoma without a BRAF mutation were randomly divided into two groups (1:1 with nivolumab at 3 mg/kg once every 2 weeks (n=210) and dacarbazine (n=208). The survival rate at 1 year was 72.9% in patients treated with nivolumab compared to 42.1% in patients who were assigned dacarbazine (HR, 0.42, P<0.001). The objective response rate was 40% with nivolumab compared to 13.9% with dacarbazine (odds ratio, 4.06; P<0.001) (79). Another randomized controlled trial was carried out between 2012-2014 on patients with advanced melanoma who progressed after ipilimumab therapy or a combination of ipilimumab and a BRAF inhibitor if they were found to be positive for a V600E mutation (80). That study assessed the role of nivolumab as a second-line treatment in the management of patients with advanced melanoma. Patients were divided into three groups in a 2:1 pattern where one group (n=272) received nivolumab at 3 mg/kg once every 2 weeks and another group (n=133) received the investigator's choice of chemotherapy (ICC), which was either dacarbazine or paclitaxel plus carboplatin (80). An interim analysis of that study found that, in the first 120 patients of the nivolumab group, 38 patients (31.7%) experienced confirmed objective responses, whereas only 5 out of the 47 patients (10.8%) receiving the ICC treatment exhibited similar responses. Subsequently, upon further analysis of that trial, it was revealed that the median overall survival rate of patients who received nivolumab was 16 months, while for those who received ICC, it was 14 months (81). The HR was 0.95, indicating that nivolumab did not improve the survival rate of patients who had ipilimumab-refractory metastatic melanoma when compared to ICC. However, nivolumab had a higher overall response rate of 27% vs. 10% for ICC, and the median duration of response was also longer for nivolumab at 32 months compared to 13 months for ICC (77). Hence, nivolumab exhibiting a higher overall response rate and a longer duration of response suggests that it may be a more effective treatment option for some patients (81). Another study was also carried out to compare the efficacy of nivolumab compared to ipilimumab as an adjuvant therapy in patients who had resected advanced melanoma. In patients with stage III or stage IV melanoma, adjuvant therapy was administered with either nivolumab (n=453) or ipilimumab (n=453) and follow-up was performed after 18 months (82). The 12-month rate of recurrence-free survival was significantly higher in the nivolumab group at 70.5%, vs. 60.8% in the ipilimumab group with a HR of 0.65 (P<0.001). It was also noted that treatment-related adverse events were 14.4% for patients treated with nivolumab and 45.9% for those treated with ipilimumab. Therefore, patients who received ipilimumab therapy experienced more severe side-effects than those who received nivolumab therapy. This suggests that nivolumab may be a more effective and tolerable treatment option for patients with stage IIIB, IIIC, or IV melanoma following surgical resection (82).

### Pembrolizumab

Pembrolizumab is a monoclonal antibody which functions by blocking the PD-1 on T-cells and allowing these T-cells to identify and kill cancer cells. Similar to nivolumab, pembrolizumab was also compared with ICC in ipilimumab-refractory melanoma. A randomized controlled study was conducted on patients with advanced melanoma and have progressed even after receiving ipilimumab and/or standard BRAF therapy (83). Patients were divided into three groups as follows: One group (n=181) received 10 mg/kg pembrolizumab, one group (n=180) received 2 mg/kg pembrolizumab, and another group (n=179) received ICC. The 6-month progression-free survival rate was found to be 38% in patients treated with pembrolizumab at 10 mg/kg (HR, 0.5 vs. ICC; P<0.0001), 34% in the 2 mg/kg group (HR, 0.57 vs. ICC; P<0.0001) and 16% in the ICC group (83). Another study was conducted by Robert *et al* (84), this time comparing pembrolizumab with ipilimumab. Patients with advanced melanoma were divided at a 1:1:1 ratio to receive pembrolizumab at 10 mg/kg once every 2 weeks or pembrolizumab at 2 mg/kg once every 3 weeks or four doses of ipilimumab at 3 mg/kg for once every 3 weeks. An interim analysis was performed which revealed that the 6-month progression-free survival of the patients treated with pembrolizumab once every 2 weeks was 47.3% (HR, 0.58 vs. ipilimumab; P<0.001), 46.4% for those treated with pembrolizumab once every 3 weeks (HR, 0.58 vs. ipilimumab; P<0.001) and 26.5% for those treated with ipilimumab (84). A final analysis revealed that the median overall survival rate was not reached in both pembrolizumab groups; however, it was noted to be 16 months in the ipilimumab group (HR, 0.68 for pembrolizumab once every 2 weeks vs. ipilimumab, P=0.0009; and HR, 0.68 for pembrolizumab once every 3 weeks vs. ipilimumab, P=0.0008) (85). Similarly, in the study by Robert *et al* (84) the 24-month overall survival rate was 55% in the group treated once every 2 weeks, 55% in the group treated once every 3 weeks and 43% in the ipilimumab group. Not only do nivolumab and pembrolizumab prolong overall survival, but they also maintain the quality of life of patients with melanoma (86,87). These findings have led to the FDA approval of pembrolizumab for ipilimumab and/or BRAF inhibitory refractory advanced melanoma. A summary of the comparison among ICIs and their outcomes in patients is presented in Table III.

## 6. Biomarkers

The prognostic marker for melanoma traditionally used is the depth of invasion and the associated mitotic count of the affected cells. With advancements being made, newer prognostic markers have been found and used. Prognostic and predictive biomarkers have gained importance, particularly in the treatment of melanoma.

### Serum biomarkers

The role of serum biomarkers in the early detection of melanoma is described in Table IV (88-106).

### Tissue markers. Prognostic markers

i) Tumor infiltrating lymphocyte (TIL) patterns are often divided into grades, such as ‘absent’, which is no presence of any lymphocytes within the tumor, ‘non-brisk’, which suggest few foci of lymphocytes within the tumor, or ‘brisk’, which is a large diffuse infiltration of lymphocytes within the tumor (107). As demonstrated by Clark *et al* (107) in 1989, as well as by others, the presence of brisk TILs in a vertical growth pattern is often associated with a favorable disease-specific survival and overall survival rate after non-brisk and absent patterns of TILs (107,108).

ii) Histotype: The majority of melanoma histotypes are not considered prognostic when looked at individually from tumor thickness, and are therefore not included in the American Joint Committee on Cancer staging system (90,109,110). However, a nodular melanoma is an independent predictor which can be used for the measurement of recurrence and its association with mortality due to melanoma (111).

iii) Digital images trained from AI: New advancements have allowed for the development of deep learning-based biomarkers, which can help to stratify the stages of melanoma into risk groups, and thus associate disease-specific survival with two independent validating cohorts to accurately predict the prognosis of patients with early-stage melanoma (112).

iv) Melanoma cell adhesion molecule (MCAM): Expressed in 80% of metastatic tumors, MCAM is a cell adhesion marker (113). Those who are positive for MCAM have significantly worse 5-year survival rates than those who are negative for MCAM, and there is an inverse association between the amount of marker expressed and survival (114,115).

v) Ki-67: Ki-67 is a unique nuclear antigen that can function as a marker for cellular proliferation during the active phase of the cell cycle (116). For melanomas who have a thickness <1 mm, the risk of metastasis increases with the expression of Ki-67 and an increased mitotic rate (117). However, with the increasing thickness of melanomas, Ki-67 can serve as a more effective prognostic marker than the mitotic rate, and is often associated with ulceration within the tumor, necrosis, higher level Clark's level of invasion, and even vascular invasion (118). In addition, with recurrent melanomas, higher values of Ki-67 exhibit an independent association with a decreased overall survival (119).

vi) Lymphatic invasion: In research on primary melanomas with a thickness >1 mm, D2-40 staining was assessed for lymphatic invasion, which is an antibody against sialoglycoprotein that selectively attaches on endothelial cells of lymphatic vessels and helps detect sentinel lymph node metastasis (120-122).

vii) Osteopontin: Overexpressed in numerous visceral malignancies, osteopontin is known as an integrin-binding protein and used as a biomarker to measure tumor progress and metastasis (123-125). It functions as an independent predictor for the prognosis of melanoma and was found to be associated with increased sentinel lymph node positivity in a cohort of 345 patients who had primary melanoma detected using immunohistochemical analysis (126).

viii) Driver mutations: It has been found that BRAF and NRAS are associated with a significantly lower melanoma-specific survival in high-risk tumors, such as a stage >2(127). NF1 mutations has also been found to be associated with a lower disease-specific survival and overall survival (128). However, further research is required to identify patients with BRAF mutations and uncover the role of BRAF mutations in directing the treatment strategy.

*Predictive marker*s. The role of predictive markers in melanoma and its clinical importance is summarized in Table V (61,129-151).

## 7. Factors affecting drug use

Therapy with nivolumab affects the frequencies of innate lymphoid cells (ILCs) in peripheral blood in patients with melanoma. The frequency, as well as the secretory activity of ILC subsets, particularly ILC2s, are affected by treatment. Albeit nivolumab was found to not effectively alter serum cytokine profiles, pro-inflammatory and angiogenic substances such as IL-1, IL-6, CCL2, CXCL8 and VEGF had levels outside the normal range in 7 of the 18 cytokines. In addition, the production of IL-5 and IL-13 was affected, which are released during parasite infections and allergic reactions (152). In malignant melanoma, type 3 ILC is suspected in tumor suppression (153). Serum levels of IL-6, CXCL8 and CCL2 in particular, surge during melanoma progression, while mature NKp44^+^ ILC3s protect against melanoma (154).

As previously demonstrated, the advancement of melanoma was comparable with aging, although the treatment outcome did not differ significantly, and there was no significant change in the survival outcomes of elderly patients as compared to young ones. Moreover, it was recommended that both age groups should be treated in similar manner (155). Primary and secondary resistance are also a key factor affecting drug use (84,156). Combination therapy with ipilimumab and nivolumab, as approved by the FDA, has been proven to be efficient (157). There is an increased incidence of melanoma among women of reproductive age. As opposed to this, postmenopausal women have a relatively low incidence of the disease, thus raising the possibility that sex hormones such as estrogen may be involved in the growth of the disease (158). As a result, estrogen levels should be considered an important biomarker in advanced melanoma. Elderly patients aged ≥65 treated with combination therapy comprising of ipilimumab and nivolumab have not exhibited a considerable difference in overall mortality. When prior exposure to ipilimumab is considered, women have a 2.82-fold increased risk of mortality as compared to prior-exposed males with ipilimumab (158).

Moderate colitis which does not require the use of intravenous steroids is consistent with an improved overall survival of patients with stage IV melanoma when treated with a single anti-CTLA-4 drug, but not with combination drugs. This holds true even after the completion of therapy (159). Multiple nonrandomized studies have shown excellent results in patients who discontinue treatment after being treated for 1-2 years and disease progression is also uncommon in the following 2-5 years of treatment termination (160-162). This is in contrast to the progression of disease of patients with non-small cell lung cancer, for whom treatment continuation led to improved results compared to treatment termination (163).

## 8. Adverse effects

The use of PD-1 inhibitors, namely nivolumab and pembrolizumab, and the anti-CTLA-4 drug, ipilimumab, has been shown to be associated with a steady regression in malignancies, including metastatic melanoma (164).

PD-1 inhibitors function in the tumor setting, while CTLA-4 inhibitors act on lymphoid tissue, resulting in a wide and different set of adverse events (165). Combination therapies with nivolumab and ipilimumab have been proven to be more effective with a response rate of 59% as compared to when used alone, with response rate of 43% for nivolumab and 15-20% for ipilimumab. Moreover, an increased response rate is associated with an increase in adverse events, resulting in an overall increase in adverse events with the combination of nivolumab with ipilimumab, as compared to nivolumab or ipilimumab monotherapy (36,62).

A CTLA-4 blockade with or without anti-PD-1 antibody produces adverse events in a dose-dependent manner (166,167). Considering that older patients are more inclined to develop rheumatologic events and female patients are also at an increased risk, the toxicity profile may vary according to age and sex (168,169).

However, as these molecules are targeted, due to the resulting immune response, an increase in the incidence of autoimmune conditions is observed; these adverse events are known as immune-related adverse events (irAE). If severe irAEs occur with one of the drugs, then it is a safe practice to re-challenge the patient with a different class of drug (165). These drugs have the following on the following systems.

### Gastrointestinal tract

Diarrhea is the most frequent irAE with incidences between 10 and 13% (164).

### Endocrine disorders

In decreasing order, the first endocrine system that is most affected by ICIs is the thyroid gland (typically hypothyroidism observed following a transient thyroiditis-induced thyrotoxicosis) followed by the rest of the endocrine organs. The median time frame from the start of the treatment to the development of thyroid symptoms, most commonly hypothyroidism, is 6 weeks, followed by pituitary (hypophysitis), adrenals (primary adrenal insufficiency) and β-cells of the pancreas (insulin deficient diabetes, analogous to type 1 diabetes) (170). These are different from the side-effects brought on by conventional cytotoxic chemotherapy or even more recent molecular-targeted medicines, which infrequently result in endocrine dysfunction (171).

### Skin disorders

Non-specific adverse events such as maculopapular rash, pruritus, psoriasiform, eczematous and lichenoid dermatosis are among the most prevalent (172,173). Compared to anti-PD-1 monotherapy, the maculopapular rash phenotype is more prevalent when CTLA-4 inhibition is implemented (21). Bullous pemphigoid, vitiligo-like skin hypopigmentation/depigmentation and alopecia are other less-common irCAEs (174,175). Although severe reactions, such as Stevens-Johnson syndrome, toxic epidermal necrolysis and drug reaction with eosinophilia and systemic symptoms are uncommon, cutaneous consequences are typically self-limiting (174-176). Early diagnosis and the administration of corticosteroids or antitumor necrosis factor-agents are the foundation of treatment algorithms for irCAEs (176,177). However, the use of corticosteroids before or after ICI initiation may result in a diminished antitumor efficacy. Anti-CTLA-4 and anti-PD1 therapy have both been associated with reports of vitiligo (178). The occurrence of skin hypopigmentation or depigmentation such as vitiligo has been linked to an extensive anticancer benefit from drug treatment in patients with melanoma. Vitiligo has been proven as a positive predictive factor in measuring the tumor response to treatment. In comparison with the general population, patients with melanoma have a 10-fold increased incidence of drug-related cutaneous hypopigmentation and depigmentation (179). Since the PD-L1:PD1 pathway mostly regulates the peripheral tolerance of melanosomal proteins (such as tyrosinase and TRP-2), the interference of PD-1 signaling may result in autoimmune vitiligo (180). This offers a reasonable explanation for the onset and durability of depigmentation in patients receiving immunotherapy.

### Lungs

Case series studies have demonstrated that patients develop organizing pneumonia, diffuse alveolar damage, acute respiratory distress syndrome (ARDS) and non-specific interstitial pneumonia, which is then managed by intravenous and oral steroids (181-185).

### Liver and kidneys

A previous meta-analysis revealed adverse effects associated with the use of anti-PD-1/PD-L1 monoclonal antibodies for malignancies with an increased incidence of pancreatitis, and increased levels of liver enzymes, such as aspartate aminotransferase and alanine transaminase, elevated creatinine levels, nephritis and renal failure (164).

## 9. Mechanisms of resistance

A myriad of ongoing clinical trials and practices have discovered multiple mechanisms leading to resistance to ICIs. More precisely, these include changes in the tumor microenvironment prohibiting T-cell interaction, tumor invasion and tumor cell destruction by effector mechanisms. The key to tumor cell destruction via effector T-cells is through the processing of tumor antigens to antigen-presenting cells. The failure of antigen-presenting components in this pathway is a major cause of resistance in melanoma (186). β2 microglobulin is an key molecule responsible for the folding and transportation of major histocompatibility complex-1 to the surface of cells. Mutations in these molecules have been noted in patients with melanoma at the time of anti-PD1 treatment failure (187). Other mechanisms responsible for limiting T-cell trafficking in the tumor microenvironment include mutations in BRAF, and the inhibition of PTEN. This leads to the increased expression of immunosuppressive molecules, such as VEGF (188). It also inhibits the migration and trafficking of effector T-cells (189). In addition to these tumor-intrinsic mechanisms, various tumor-extrinsic mechanisms also play a role in the development of resistance to ICIs. These include the development of new inhibitory checkpoints, immunosuppressive cytokines and molecules in the tumor microenvironment suppressing immune cell function. One such example is the production of transforming growth factor β (TGF-β) by tumor cells. TGF-β is an immunosuppressive cytokine that functions by stimulating Tregs and inhibiting the cytotoxicity of effector T-cells (190).

To summarize, understanding and investigating the potential mechanisms that lead to resistance to ICIs is crucial in developing effective strategies to guide therapy. Further studies are required to identify new mechanisms and develop targeted therapies to improve the clinical outcome of patients undertaking immunotherapy.

## 10. Emerging newer therapeutic strategies: Targeting tumor metabolic dependencies

Tumor cells sustain themselves by utilizing altered metabolic pathways by using nutrients, such as glucose, tryptophan and arginine to produce toxic metabolites such as adenosine, lactate and kynurenine (191,192). Such toxic metabolites produce an unfavorable environment for the antitumor cells to function resulting in increased expression of immune checkpoints and expansion of Tregs (193).

The mechanism that tumor cells use is the mutation in the myelocytomatosis oncogene (MYC) and PI3K/AKT/mammalian target of rapamycin (mTOR) signaling pathways. The increased expression of hypoxia-inducible factor-1-α leads to the overexpression of the PI3K/AKT/mTOR pathway, as well as glucose transporters such as glucose transporter 1, leading to increased glucose consumption and acidification of the tumor microenvironment (194,195). As hypoxia is generated, glucose depletion occurs and increased toxic waste is produced within the tumor microenvironment, resulting in the inhibition of tumor antigen presentation by APCs (196). Thus, there is an overall decrease in the antitumor immune response by T-effector, macrophages or NK cells, while pro-tumor immune cells such as Tregs proliferate to increase the expression of inhibitory checkpoint ligand PD-1 on immune cells, inhibiting the antitumor immunity (197). With the advancement of technologies, newer therapeutic strategies that target the immunosuppressive tumor microenvironment generated by tumor cells may be developed to reprogram the behavior of immune cells, leading to an improved efficacy in terms of the treatment response.

One of the important T-cellular processes is the activation of the PI3K pathway, which plays a vital role in proliferation and differentiation. Monotherapy, which inhibits the PI3K pathway, has not yielded any significant results in the treatment of cancer; however, combining PI3K inhibitors and the PD-1-PDL1 blockade has shown some notable results (198). The loss of PTEN, which is a PI3K-inhibiting tumor suppressor often mutated in tumor cells, results in the uncontrolled growth of tumor cells and escapes the immune destruction imposed on it. As previously demonstrated, when mice with PTEN-null melanoma were treated *in vivo* with the PI3Kβ inhibitor, GSK2636771, this resulted in a decreased AKT phosphorylation and the activation of mTOR targets. Additionally, when it was combined with an anti-PD1 antibody, it markedly improved the survival and increased immune response with reduced tumor cell mass (199). With such promising results, a number of newer anti-PI3K medicines are being developed and tested to increase efficacy (NCT01390818). Despite this, more novel promising approaches are needed to prove the success of combining anti-PI3K drugs with ICIs in the treatment of melanoma (200).

## 11. Conclusion and future perspectives

The development of ICIs and targeted therapies has played a crucial role in revolutionizing the management of melanomas by improving the overall and progression-free survival. Although both of these therapies have advantages and disadvantages, combination therapy (ICI + ICI, or ICI + targeted therapies) has been found to be more effective in improving patient outcomes. However, there is limited literature available regarding combination therapies and different types of potential combinations. There are also insufficient data on patients and their responses to draw sufficient conclusions. The development of drug-related adverse effects with the use of combination therapies is also a debatable question. However, when developing newer ICIs to achieve a more effective response, a focus should certainly be placed on the integration of nanotechnology or antibody engineering. Through these, one can increase drug delivery to a specific target and thus increase overall response. In addition, focusing on epigenetic modulation and developing ICIs that target those changes can enhance the responsiveness of ICIs.

There may be concerns regarding resistance to ICIs in patients with melanoma. Some patients may have resistance to certain ICIs from the beginning or may develop them as an acquired resistance with subsequent treatment after progression of a tumor with clinical benefit. Further treatment decisions shall be made on the basis of evaluation of the tumor and factors related to the patient, focusing on targeted therapeutic drugs, other immunotherapy drugs, cellular therapies, intralesional therapies, or chemotherapy. It is important to tailor ICI treatment based on an individual's genetic makeup and tumor characteristics to decrease the resistance. The early identification of tumor biomarkers can predict future responses to particular ICIs and may help to select a personalized treatment strategy. Furthermore, with the use of tumor metabolic pathway inhibitors in combination with ICIs, targeting signaling pathways and immune responses can be better used to overcome potential resistance to ICIs than when used alone.

Trials are being conducted on newer inhibitory immune checkpoint targets, as well as certain inhibitory targets beyond immune checkpoints. These include LAF-3, TIM-3, B7-H3 and B7-H4, CD73, etc. which are immune checkpoints, and CEACAM1, CEACAM5/6, CCL2/CCR2, etc. which are other inhibitory targets (201). It is essential to maintain enrollment in clinical trials so that newer ICIs, additional inhibitory treatments, combination therapy, and mechanisms of resistance and methods of overcoming the resistance can all be further investigated.

In conclusion, with the increasing incidence of melanoma over the past two decades, managing it with different treatment modalities has become cumbersome. With the limited effectiveness of the traditional approach using chemotherapy and immunotherapy, the role of newer treatment modalities should be given equal emphasis. Novel approaches using ICIs have been a revolution in the therapeutic approach by unleashing the immune system's ability to recognize and eliminate cancer cells. Ipilimumab, nivolumab and pembrolizumab have been shown to lead to a substantial improvement in the overall survival of patients with advanced melanoma, particularly in high-risk metastatic melanoma compared to traditional therapies. However, with ICIs, it is paramount to monitor any side-effects, and to ensure the optimal outcome is achieved using personalized treatment approaches.

## Figures and Tables

**Table I:** Summary of the results of the CheckMate and RELATIVITY047 trials performed on the combination of immune checkpoint inhibitors for the treatment of melanoma.

Study name	Treatment group	Objective response rate (%)	Median PFS	5-year PFS rate	Median overall survival	5-Year overall survival rate	(Refs.)
CheckMate 064	Nivolumab followed by ipilimumab	41	-	-	76%	-	(56)
CheckMate 064	Ipilimumab followed by nivolumab	20	-	-	54%	-	(56)
Checkmate 067	Nivolumab-ipilimumab	58	11.5 months	36%	36.9 months	52%	(57)
Checkmate 067	Nivolumab	45	6.9 months	29%	-	44%	(57)
Checkmate 067	Ipilimumab	19	2.9 months	8%	19.9 months	26%	(57)
RELATIVITY-047	Nivolumab-relatlimab	47.7	10.1 months	-	-	-	(54)
RELATIVITY-047	Nivolumab	36	4.6 months	-	-	-	(54)

PFS, progression-free survival.

**Table II:** Summary of results obtained from a previous meta-analysis of clinical trials conducted by Pradeep *et al* (58), which compared the efficacy and safety of ICIs between monotherapy and combined ICI therapy in advanced melanomas.

Outcome [(Refs.); as in the meta-analysis by Pradeep *et al* (58)]	Comparison	Effect size in terms of HR or RR (95% CI)	P-value
Overall survival (52-56)	Nivolumab with ipilimumab vs. monotherapy	HR, 0.65 (0.53-0.79)	<0.0001
	Nivolumab with ipilimumab vs. nivolumab alone	HR, 0.84 (0.71-0.99)	0.04
	Nivolumab with ipilimumab vs. ipilimumab alone	HR, 0.54 (0.48-0.62)	<0.00001
Progression-free survival (53,55-58)	Nivolumab with ipilimumab vs. monotherapy	HR, 0.48 (0.38-0.60)	<0.0001
	Nivolumab with ipilimumab vs. nivolumab alone	HR, 0.68 (0.49-0.94)	0.02
	Nivolumab + ipilimumab vs. ipilimumab alone	HR, 0.42 (0.37-0.47)	<0.00001
Overall response rate (52,54-57,59)	Nivolumab with ipilimumab vs. monotherapy	RR, 2.15 (1.63-2.84)	<0.00001
	Nivolumab with ipilimumab vs. nivolumab alone	RR, 1.32 (1.22-1.43)	<0.00001
	Nivolumab with ipilimumab vs. ipilimumab alone	RR, 3.09 (2.74-3.50)	<0.00001

In the meta-analysis by Pradeep *et al* (58), overall survival, progression-free survival and objective response rates were the outcomes studied. HR, hazard ratio; RR, relative risk.

**Table III:** Individual immune checkpoint inhibitors with the various outcomes affecting clinical decisions regarding their use.

ICI drug	Compared drug	Outcome	(Refs.)
Ipilimumab	Ipilimumab (3 mg/kg) (n=137) vs. Gp100 (n=136) vs. ipilimumab (3 mg/kg) with gp100 (n=403)	Median overall survival (months): 10.1 vs. 6.4 vs. 10.0	(20)
	Ipilimumab (10 mg/kg) with dacarbazine (n=251) vs. dacarbazine (n=251)	Median overall survival (months): 11.2 vs. 9.1	(76)
	Ipilimumab (10 mg/kg) adjuvant (n=475) vs. placebo (n=476)	Recurrence-free survival (%): 40.8 vs. 30.3	(77)
	Ipilimumab (10 mg/kg) (n=365) vs. ipilimumab (3 mg/kg) (n=362)	Median overall survival (months): 15.7 vs. 11.5	(78)
Nivolumab	Nivolumab(3 mg/kg) once every 2 weeks (n=210) vs. dacarbazine (n=208)	1-Year survival rate (%): 72.9 vs. 42.1; objective response rate (%): 40 vs. 13.9	(79)
	Nivolumab (3 mg/kg) (n=272) vs. ICC (dacarbazine or paclitaxel plus carboplatin) (n=133)	Median overall survival rate (months): 16 vs. 14; overall response rate (%): 27 vs. 10	(80)
	Nivolumab (n=453) vs. ipilimumab (n=453)	12-Month recurrence-free survival rate (%): 70.5 vs. 60.8; treatment-related adverse events (%): 14.4 vs. 45.9	(82)
Pembrolizumab	Pembrolizumab (10 mg/kg) (n=181) vs. pembrolizumab (2 mg/kg) (n=180) vs. ICC (paclitaxel plus carboplatin, paclitaxel, carboplatin, dacarbazine, or oral temozolomide) (n=179)	6-Month progression-free survival rate (%): 38 vs. 34 vs. 16	(83)
	Pembrolizumab (10 mg/kg once every 2 weeks) (n=279) vs. pembrolizumab (2 mg/kg once every 3 weeks) (n=277) vs. four doses of ipilimumab (3 mg/kg once every 3 weeks) (n=278)	6-Month progression-free survival rate (%): 47.3 vs. 46.4 vs. 26.5; median overall survival rate (months): NA vs. NA vs. 16; 24-month overall survival rate (%): 55 vs. 55 vs. 43	(85)

ICC, investigator's choice of chemotherapy; NA, not available.

**Table IV:** Role of serum biomarkers in melanoma.

Biomarker	Use	Type	Information (Refs.)
LDH	Prognostic and predictive	Protein-based	The AJCC staging scheme includes blood LDH as an independent prognostic biomarker for metastatic melanoma. A univariate analysis revealed that an increased amount of LDH was associated with a lesser response to anti-PD-1 medications in patients who received anti-PD-1 monotherapy (88). It was previously found that patients with elevated LDH and NLR levels, despite receiving ICIs, had poor a OS and PFS (89). Elevated serum LDH levels increased the disease stage of patients with metastasis to stage IV M1c (1) metastatic disease, which included individuals with metastases to the lungs, as well as to other visceral sites apart from the central nervous system (90,91).
S100	Prognostic	Protein-based	S100B levels in serum are affected by the tumor load, and higher values of S100B may be used as a prognostic biomarker and a sign of disease progression (92-94). It was previously found that the discriminative ability with serum S100B levels for detecting disease relapse was much higher than that of serum LDH (95).
miRNA	Prognostic	Genomic	The role of miRNAs as post-transcriptional inhibitors of translation has been substantially investigated (96-100). It has been discovered that these small non-coding RNAs are detectable in exosomes in serum (101). It has been demonstrated that aberrant levels of expression of certain miRNAs are associated with the stage and recurrence of disease (102,103). miRNAs are crucial in the deregulation of a number of oncogenic pathways in uveal melanoma, which can facilitate the metastatic spread of disease. The study by Wróblewska *et al* (104) found that it may be beneficial to use differentially expressed miRNAs as a biomarker for the evaluation of the risk of metastasis in patients with uveal melanoma.
ctDNA	Prognostic	Genetic	It has been demonstrated that circulating tumor DNA testing may detect BRAF V600e mutations, which is helpful for determining how effectively the BRAF/MEK inhibitor therapy works. Imaging scan measurements of disease progression and treatment effectiveness have been found to be associated with the amount of circulating tumor DNA (105,106). Both univariate and multivariate analyses have revealed an association between ctDNA detectability and a shorter OS. Patients with distant metastases (79%) were more likely to have ctDNA detected than those with no distant metastases or only intracranial tumors (32%). Elevated protein S100 and CRP levels were more closely linked to detectable ctDNA than LDH (104).

AJCC, American Joint Committee on Cancer; LDH, lactate dehydrogenase; PD-1, programmed cell death protein 1; NLR, neutrophil-lymphocyte ratio; ICI, immune checkpoint inhibitor; OS, overall survival; PFS, progression-free survival; miRNA, microRNA; ctDNA, circulating tumor DNA.

**Table V:** Role of predictive biomarkers and their clinical relevance.

Type	Biomarker	Clinical relevance (Refs.)
Tumor intrinsic	TMB/neoantigen profile	Tumors with a higher TMB are potentially more responsive to ICIs; a reason for this may be the fact that these tumors express more neoantigen and hence, may be recognized easily as a target by T-cells (129). With the increasing evidence of the ability of TMBs to predict the response to immunotherapy in melanoma, various studies have been conducted (130,131). In a previous study, 321 patients with melanoma were treated with ICIs and it was found that with a higher TMB, one could predict an increased survival following treatment (132).
	Driver mutations	Immunotherapy was found to be more effective in patients with activating NRAS genes than in those without such mutations. According to the CheckMate 067 study, those patients with BRAF-mutation in melanoma had a 4-year overall survival rate of 62% with combination therapy with ipilimumab and nivolumab, when compared to only 50% with nivolumab alone and 33% with ipilimumab alone (61,133).
Tumor microenvironment	MHC	Longer overall survival, a greater response rate, and an increased infiltration of CD4^+^ and CD8^+^ T-cells in the tumor microenvironment were all associated with elevated levels of MHC-II expression. Conversely, ipilimumab treatment was found to be associated with a higher risk of disease progression when MHC-I expression was lower (<30%), with a 100% negative predictive value (134-136).
	HLA supertypes	Two HLA supertypes were identified and linked to CTLA-4 inhibition therapeutic outcomes: HLA-B44 was associated with a longer survival, while HLA-B62 was associated with a shorter survival (137).
	PD-L1	PD-L1 is a complex biomarker to study since it is regulated by several pathways, which is vulnerable to high sampling error, and expressed on numerous immune cells in the microenvironment (138). Treatment with PD-1 inhibitors has been demonstrated to be successful in both patients with and without PD-L expression; however, the quality of immunohistochemical staining for PD-L1 is not dependable for clinical use (139,141).
	Immune metabolism	ICI-sensitive melanomas exhibited more oxidative phosphorylation and lipid metabolism than ICI-resistant tumors using high resolution mass spectrometry. In addition, elevated lipid metabolism increases antigen presentation, which may be the reason why ICI-sensitive cancers have a better response to therapy (142).
	TIL/TCF-7	It has been demonstrated that TCF-7, a transcription factor, promotes a central memory stem-like state, and that TCF7-expressing CD8^+^ cells have the ability to self-renew and differentiate into effector cells (143,144). TCF-7-positive cells appear to be the ones that multiply following anti-PD1 therapy (145). According to this finding, TCF-7 expression has been linked to successful clinical results in patients with melanoma receiving ICIs (146). In light of this, further research is required to establish the role of TCF7 expression as a predictive biomarker in patients with melanoma who are being treated with ICIs and its role as a therapeutic target.
	GEP	Research has been performed to identify a gene expression profile (GEP) that can predict the response to pembrolizumab. It was discovered that the 18-gene profile could identify aspects of the tumor microenvironment that are pertinent to predicting the clinical outcome of pembrolizumab that are independent of tumor type. This indicates that GEPs, such as the 18-gene profile, can be utilized to predict how different types of cancer will respond to PD-1/PD-L1 inhibitors (147-149).
	IPRES	The IPRES transcriptional signature is a peculiar pattern of gene expression that is present in tumors which are resistant to anti-PD-1 medications. Increased activity in genes is related to an increase in transition through the mesenchyme, helping in cell adhesion, extracellular matrix remodeling, increase in angiogenesis, and wound repair which are the hallmark of this pattern. The response rate to anti-PD-1 therapy can be enhanced with the identification of transcriptomic features that are associated with anti-PD-1 resistance, which may suggest the mitigation of IPRES-related biological process and eventually, an enhance response rate to treatment (147)
Host factors	Gut microbiome	Patients with high-fiber diets were 5-fold more likely than those with low-fiber diets to respond to anti-PD-1 therapy. In addition, regardless of the type of antibiotic, indi viduals who had received antibiotics for >30 days prior to commencing ICI treatment suffered worse outcomes. A past but not concurrent antibiotic treatment has an impact on the gut microbiome's ‘activating’ response to ICIs (150).
	Stress	A solid tumor line was grafted into mice, and they were then treated with an anti-PD-1 monoclonal antibody. Subsequently, they were made to experience social defeat stress, and compared to the mice who did not experience this type of stress; the mice who were socially defeated with stress responded to PD-1 inhibition less effectively (151).
Others	AI	According to recent research, pre-treatment computerized H&E slides can be used to artificially predict the probability of a response to ICIs (112).

TMB, tumor mutational burden; ICI, immune checkpoint inhibitor; MHC, major histocompatibility complex; HLA, human leukocyte antigen; CTLA-4, cytotoxic T-lymphocyte-associated antigen-4; PD-L1, programmed cell death protein ligand 1; TCF-7, transcription factor 7; IPRES, innate anti-PD1 resistance; AI, artificial intelligence; H&E, hematoxylin and eosin.

## Data Availability

Not applicable.
